# Air-like plasmonics with ultralow-refractive-index silica aerogels

**DOI:** 10.1038/s41598-019-38859-2

**Published:** 2019-02-19

**Authors:** Yeonhong Kim, Seunghwa Baek, Prince Gupta, Changwook Kim, Kiseok Chang, Sung-Pil Ryu, Hansaem Kang, Wook Sung Kim, Jaemin Myoung, Wounjhang Park, Kyoungsik Kim

**Affiliations:** 1 School of Mechanical Engineering, Yonsei University, 50 Yonsei-ro, Seodaemun-gu, Seoul, 03722 Republic of Korea; 2 Technology Collaboration Team, LG Display Co., Ltd., Gyeonggi-do, 413-811 Republic of Korea; 3 Department of Materials Science and Engineering, Yonsei University, Seoul, 03722 Republic of Korea; 4 Department of Electrical Engineering, University of Colorado, Boulder, Colorado, USA

## Abstract

The coupling of the surface plasmon near-field into the sensing medium is key to the sensitivity of surface plasmon-based sensing devices. A low-index dielectric is necessary for the sensing medium to support a highly-penetrating surface plasmon evanescent field that extends well into the dielectric medium. The air-like refractive index, *n*, of an aerogel substrate provides another dimension for ultralow-index plasmonic devices. In this paper, we experimentally observed an angular surface plasmon resonance dip at 74° with the ultralow-index aerogel substrate, as was expected from theory. We also demonstrated the comparatively high-sensitivity surface plasmon resonance wavelength, *λ*, while the change in Δ*λ*/Δ*n* with different substrates was studied in detail. A 740 nm-period metal grating was imprinted on aerogel (*n* = 1.08) and polydimethylsiloxane (PDMS; *n* = 1.4) substrates. The ultraviolet–visible–near-infrared spectra were observed in the reflection mode on the grating, resulting in sensitivities of 740.2 and 655.9 nm/RIU for the aerogel and PDMS substrates, respectively. Numerical simulations were performed to understand the near-field of the surface plasmon, which demonstrated resonances well correlated with the experimentally observed results. The near-field due to excitation of the surface plasmon polaritons is observed to be more confined and to penetrate deeper into the sensing medium when a low-index substrate is used.

## Introduction

A surface plasmon polaritons (SPP) induce strong confinement and intense optical fields, resulting in high-performance label-free biosensing platforms. Typically, the SPP effect is produced and manipulated by controlling the geometry, materials and optical environment at the interface between the metal and the dielectric materials. Further, the spectral position of the SPP is usually detected as a peak in the optical absorption, reflection or extinction spectra. The SPP effects are strongly dependent on the permittivity of the surrounding medium, and thus an increase of the refractive index-sensing medium (i.e., analyte) in the close vicinity of the sensing platform produces a red-shift of the resonance position, which offers a refractive index sensor based on plasmonics^1–13^.

In plasmonic refractive sensors made of metallic nanostructures on a dielectric substrate, an antisymmetric SPP mode is generated across the metal thin film and the optical field is mainly concentrated at the substrate/metal interface^14,15^. By changing the refractive index of the substrate we can control the electromagnetic field distribution of the antisymmetric mode, where higher refractive-indexed substrates produce a larger concentration of the field in the substrate/metal interface. In contrast, a reduced substrate refractive index moves the confined electromagnetic fields from the substrate side towards the sensing region. As a result, the bulk sensitivity is able to be enhanced via the more concentrated near-fields of excited SPP in the analyte by using substrates with lower refractive indices. Using the low-index substrate of Teflon™ (*n*≃1.31), for example, enhanced plasmonic sensitivities have been observed using nano-hole or nano-slot structures^16^.

To realize even lower refractive indices, thin films (*n* = 1.0–1.3) have been developed using glancing-angle-deposited nanowires, sponge-like block copolymers, and porous silica^17–20^. However, the applications of these ultralow-index films have mainly been aimed at an enhanced antireflective surface via the index-matching condition between two media at the transmitting interface; satisfying, for example, *n* = 1.21 at the air/glass interface^18,21–24^. Among the ultralow-refractive index materials, silica aerogel has the lowest “air-like” refractive index of less than 1.1 and a high optical transparency over 90%^25,26^. The attenuation coefficient of the aerogel has been measured as 0.069 mm^−1^ for the broadband wavelength of 400–700 nm^27,28^. Moreover, aerogels can be manufactured using large-scale, low-cost and high-throughput processes. Herein, we apply a large-area porous silica aerogel to a refractive index sensor template substrate to obtain enhancement of the plasmonic sensitivity.

The sensitivity of a plasmonic sensor is represented by changes in the plasmonic resonant peak associated with the change in the refractive index of the surrounding medium. A number of studies have improved sensitivity by changing the plasmonic structures^29,30^, but the application of these studies is limited because of the amount of effort necessary to create the structure. Therefore, research is needed to improve the sensitivity by a simple method. Several studies have reported that the refractive index of the substrate will affect the sensitivity of a plasmonic structure, whereby a substrate whose refractive index is close to that of air will exhibit a significantly increased sensitivity.

In this study, we present the strong impact that the substrate has on the bulk, propagating SPP resonance sensor sensitivities. We show that a reduction of the substrate refractive index leads to an increase in the sensitivity. This improvement is achieved despite the fact that the peak position of the resonance is blue-shifted when the substrate refractive index is reduced, owing to the change of the electromagnetic field distribution that is concentrated in the analyte region for ultralow-index substrates. We experimentally observed an angular SPP dip at 74° for the ultralow-index aerogel substrate (*n* = 1.08), which is consistent with theory. We compared two cases using substrates with different refractive indices; namely an aerogel (*n* = 1.08) and a polydimethylsiloxane (PDMS; *n* = 1.40) substrate. A one-dimensional nanograting structure is patterned on the substrate by imprinting with a rewriteable compact disc (DVD-RW) recording surface pattern. By varying the refractive index of the sensing analytes, the SPP resonance wavelength was measured for a silver grating with the aerogel and the PDMS substrates. The change in resonance wavelength versus refractive index unit (RIU) is more prominent for the ultralow-index substrate (aerogel) than the higher-index substrate (PDMS). We experimentally observed the RIU values for the aerogel and PDMS as 740.2 and 655.9 nm/RIU, respectively. Our numerical simulation using COMSOL software showed that surface plasmon near-field couples well into the analyte for the lower-index substrate, identifying it as a good candidate for sensing. The experimental results are well correlated with the simulated results.

### Experimental Section

#### Fabrication of aerogel nanograting

**Figure 1:**
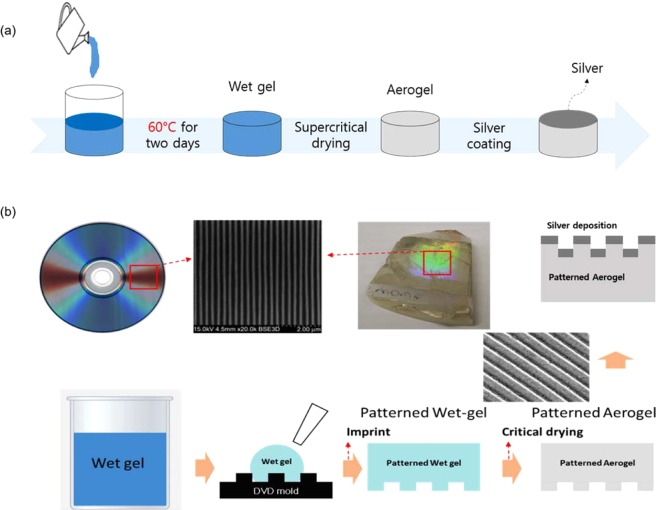
(**a**) Fabrication procedure for the silver-coated silica aerogel structure: the silica wet gel was prepared by mixing MTMS, surfactant, and acetic acid. The aerogels were obtained by drying the wet gel with supercritical carbon dioxide at 80 °C and 13.5 MPa. A thin silver film was deposited on the aerogel by electron-beam evaporation to create a plasmonic nanogap-based surface-enhanced Raman scattering active substrate. (**b**) Fabrication flow chart of the localized plasmonic sensor with aerogel.

A schematic of the fabrication process used for the bulk aerogels is presented in Fig. 1(a). Into 7.0 g of 10 mM acetic acid solution was mixed 4.76 g of methyltrimethoxysilane (MTMS), 0.5 g of urea, and 1.1 g of nonionic surfactant poly(ethylene oxide)-blockpoly(propylene oxide)-block-poly(ethylene oxide) triblock copolymer (Pluronic F127)^25–27^. After continuous stirring for 30 min at room temperature, the mixed solution was slowly poured into a Petri dish. For a solid gelation, the mixed solution was placed in a closed vessel and then into an oven at 60 °C for 2 days. To remove the surfactant and any unreacted species, etc., that may be present in the mesopores of the silica chain network, the gel was immersed in distilled water for 1 day and then in isopropyl alcohol (IPA) at 60 °C for 2 days to exchange the solvent with 2-propanol. Finally, the aerogel was obtained after removing the IPA by drying the gel in a chamber with the supercritical condition of carbon dioxide at 80 °C and a pressure of 13.5 MPa. To apply these aerogels with air-like refractive indices into plasmonics, we deposited a 60 nm-thick silver layer on the surface of the aerogel using an electron-beam evaporator.

Figure 1(b) shows the fabrication flow of the refractive index sensor with an aerogel grating structure based on SPP resonance applications. Because lithography fabrication techniques based on focused ion beams or electron beams are not suitable for practical applications, we employed a simple nanoimprinting method using a mold comprising a DVD-RW recording surface possessing a 740 nm-period grating structure, which is large-scale, low-cost, and easily available. To create the patterned aerogel substrate, we injected wet gel into the DVD-RW mold. After solidifying and drying the wet gel, a 60 nm-thick silver layer was deposited on the patterned surface of the aerogel substrate using an electron-beam evaporator. As a reference sample, we also made a PDMS substrate with the same grating structure using a similar method.

#### Geometric sample morphologies

**Figure 2:**
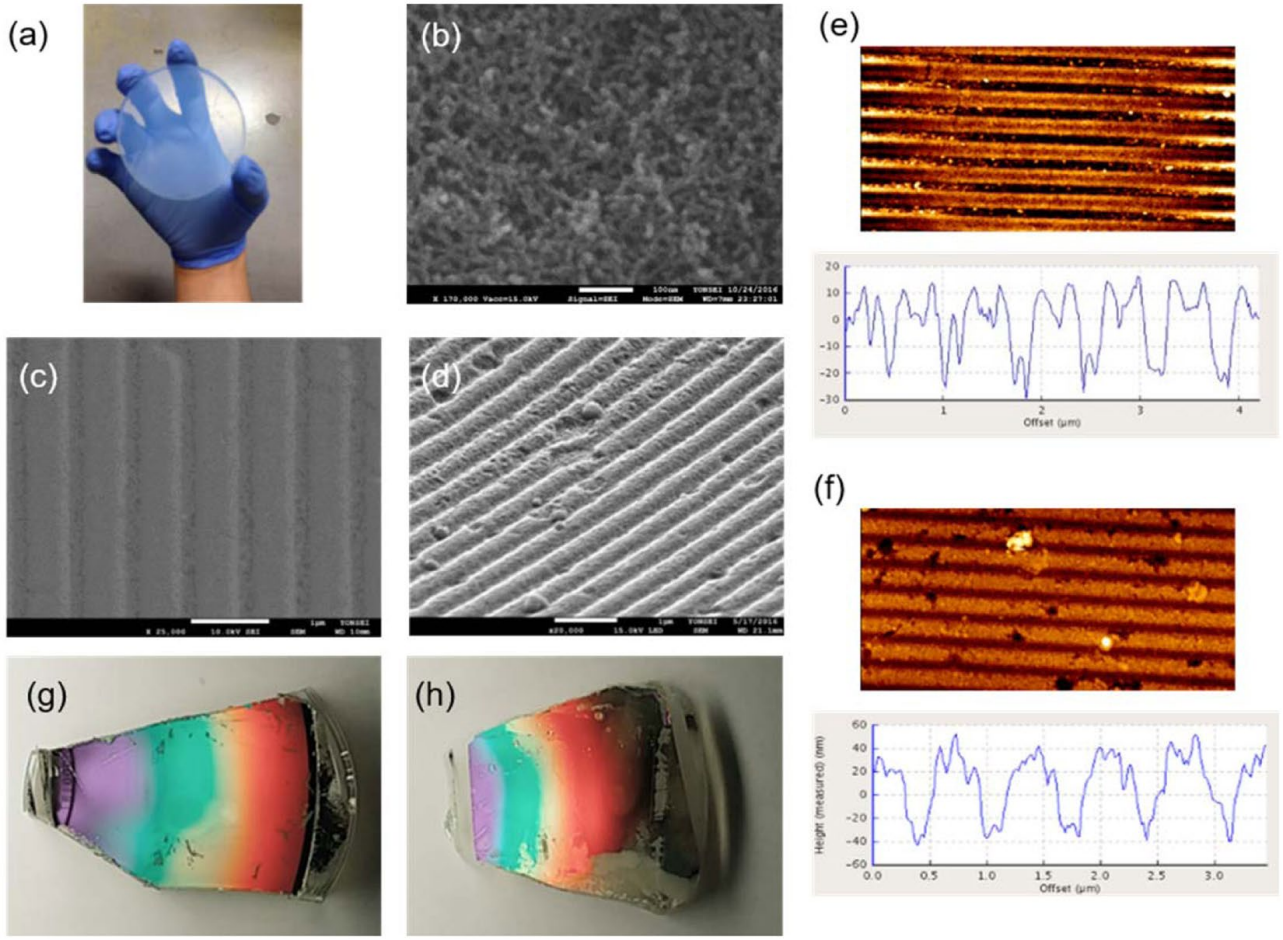
(**a**) Photograph and (**b**) SEM image of the bulk aerogel. (**c**,**d**) Tilt SEM images of the DVD-RW-patterned (**c**) PDMS (*n* = 1.40) and (**d**) aerogel (*n* = 1.08) substrates coated with Ag. (**e**,**f**) AFM data of DVD-RW-patterned (**e**) PDMS and (**f**) aerogel substrates coated with Ag. (**g**,**h**) Photographs of the DVD-RW-patterned (**g**) PDMS and (**h**) aerogel coated with Ag. The Ag films were all 60 nm thick.

Figure 2(a) presents an optical photograph of the silica aerogel. The bare and Ag-coated aerogel surfaces were characterized by scanning electron microscopy (SEM) and atomic force microscopy (AFM). As shown in the SEM image (Fig. 2(b)), the silica aerogel surface has a highly nanoporous structure produced by the cross-linked silica chain network^25,27^. The typical mean pore size and porosity of the fabricated silica aerogels are 60 nm and 84%, respectively^25,27^. To create a plasmonic structure with an air-like substrate, 60 nm-thick Ag was coated on the top of the aerogel surface, which exhibited a well-connected domain as a planar layer. We fabricated these Ag(60 nm)-coated grating structures with a 740 nm period using both aerogel and PDMS substrates. The SEM tilt images of the Ag(60 nm)-coated grating structures of the aerogel and PDMS substrates are shown in Fig. 2(c,d), respectively, while Fig. 2(e,f) respectively show the AFM images and corresponding intensity line scans and Fig. 2(g,h) respectively show optical photographs of the Ag-deposited grating structures of the aerogel and PDMS substrates.

## Results and Discussion

### Optical properties of aerogel

Using Snell’s law, we experimentally measured the refractive index of our aerogel sample. The resultant refractive index of silica aerogel is 1.08 in the visible wavelength region, which is close to air^26^. The experimental set up and method are described in detail in ref.^27,28^. To confirm the ultralow index value by total internal reflection scheme, we coupled a laser beam from air into the facet of an aerogel slab with the varying incidence angle. Total internal reflection was observed beyond the critical angle of 67.1°, which is consistent with the ultralow refractive index of 1.08.

### Measurement of SPP resonance angle for air-like aerogel

**Figure 3:**
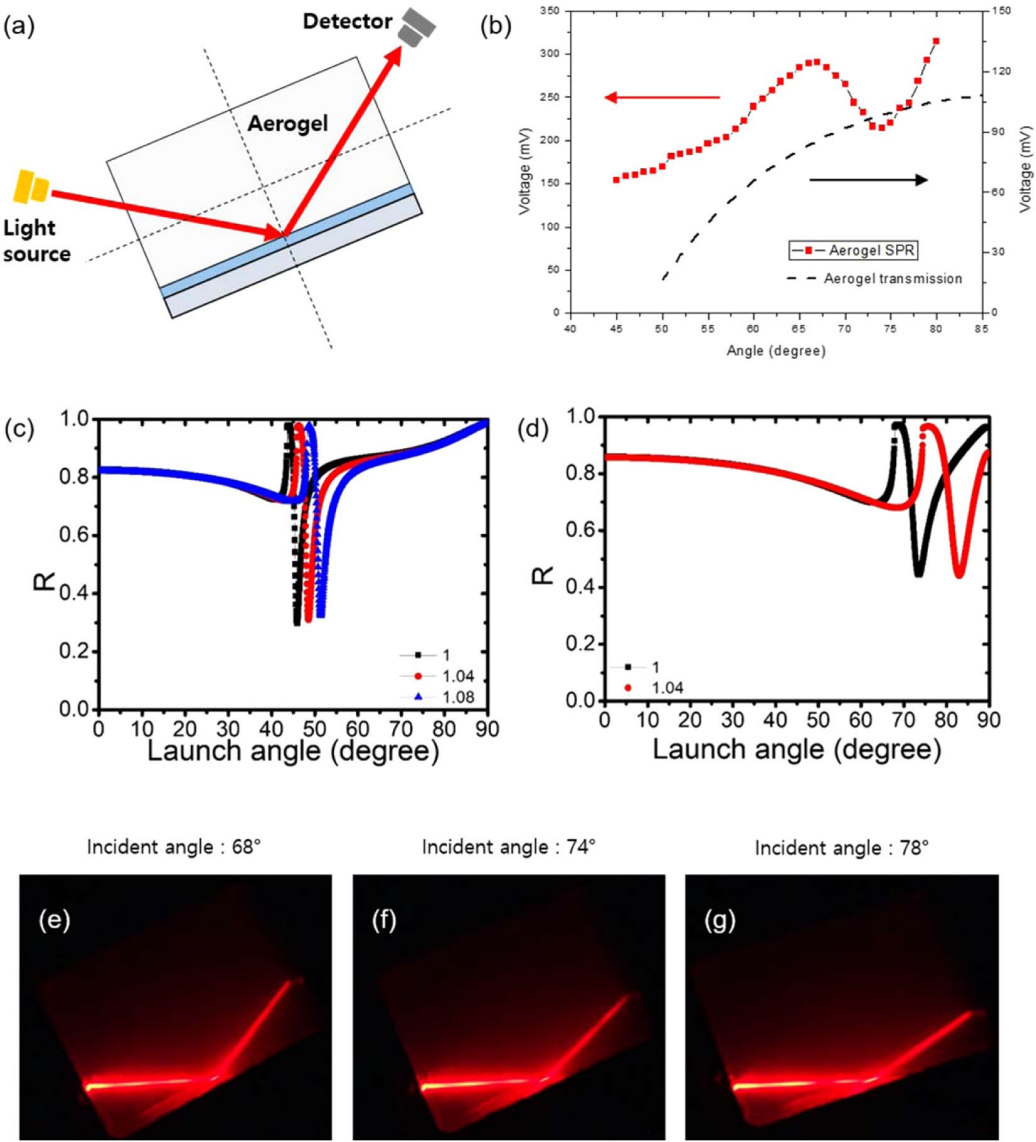
(**a**) Schematic of the aerogel-based surface plasmon resonance setup. (**b**) Experimentally measured reflection data for the SPP resonance of an aerogel coated with 30 nm-thick Ag as a function of incident angle. (**c**,**d**) Fresnel reflection data, *R*, for a bulk- SPP resonance refractive index sensor on (**c**) glass (*n* = 1.45) and (**d**) aerogel (*n* = 1.08) substrates. (**e**–**g**) Image of the incident laser beam at an angle of (**e**) 68°, (**f**) 74° and (**g**) 78°.

To investigate the plasmonic resonances of the silica aerogel (3.0 × 4.0 × 0.5 cm^3^) with an air-like refractive index, we employed the scheme, shown in Fig. 3(a), in which angle-dependent SPP resonances are typically characterized in an aerogel coated by a metallic layer in the bottom. We used the refractive indices of the materials at 633 nm as n_silicon_ = 1.45, n_aerogel_ = 1.08, and ε_Ag_ = −15.9317 + 1.0766*i*, respectively. For a 30 nm-thick silver layer on a aerogel (n = 1.04), an experimentally characterized SPP resonances with varying angles is shown in Fig. 3(b) and corresponding calculated angle dependent SPP resonance is shown in Fig. 3(d). To characterize the angle sensitivity, we also calculated the SPP angle by changing the sensing medium virtually to n = 1, 1.04 and 1.08 using a glass (n = 1.45) coated with 30 nm silver (Fig. 3(c)).

To calculate the reflectivity, *R*, of the incident radiation at the insulator-metal-insulator tri-layer thin film, we used the Fresnel equation for transverse-magnetic polarized light, given as

1 $$R={|{r}_{012}^{p}|}^{2}={|\frac{{r}_{01}^{p}+{r}_{12}^{p}{e}^{2i{k}_{z1}d}}{1+{r}_{01}^{p}\times {r}_{12}^{p}{e}^{2i{k}_{z1}d}}|}^{2}$$

$${r}_{ik}^{p}=\frac{\frac{{k}_{zi}}{{\varepsilon }_{i}}-\frac{{k}_{zk}}{{\varepsilon }_{k}}}{\frac{{k}_{zi}}{{\varepsilon }_{i}}+\frac{{k}_{zk}}{{\varepsilon }_{k}}}$$

where *i*, *k* = 0, 1, 2.

The reflectivity was calculated at the dielectric (i.e., analyte)/silver/(aerogel or PDMS) interface, when the light was launched at an angle of incidence *θ*_0_. The value of *k*_*zi*_ = (*ε*_*i*_*k*_0_^2^−*k*_*x*_^2^)^1/2^ was inserted into Eq. (1) for the three media *i* = 0, 1 and 2 correlating to the dielectric substrate (*i* = 0), the silver (*i* = 1) and the superstrate analyte (*i* = 2). The value of *k*_*x*_ was calculated using the momentum matching condition of an in-plane wave-vector of incident light at a one-dimensional thin film in the Kretschmann configuration for SPP excitation, given as *k*_*x*_ = (*ε*_0_)^1/2^ *k*_0_ sin*θ*, where *k*_0_ = 2π/*λ*, (*m* = 1,2,3…). The value of the dielectric permittivity of silver (*ε*_1_ = −15.9317 + *i*1.0766) was taken from the optical observations of Palik^31^. Three superstrates of air, aerogel and glass were used for the calculation, whose dielectric permittivity values (*ε*_2_) were 1, 1.04 and 1.08, respectively. The dielectric permittivity values of the substrate (*ε*_0_) for aerogel and glass were 1.08 and 1.45, respectively. The reflectivity was calculated at an incidence angle range *θ* = 0–90° at the wavelength of 633 nm. The plots of the reflectivity versus incidence angle are shown in Fig. 3(c,d) for the aerogel and glass substrate, respectively, which show values that correspond well with experimental data.

To experimentally verify the plasmonics of air-like aerogels, we used typical Kretschmann configuration set up for SPP resonance angle measurement. We illuminated a TM-polarized 633 nm laser light into a 30 nm-thick silver coated rectangular aerogel substrate in the first rotating stage and the photodetector in the second rotating arm. The rotation of both arms was synchronous, making the same angle with the vertical. The reflected light intensity was measured as a function of the incidence angle at the aerogel-metal interface. Figure 3(b) shows the experimentally observed reflection intensity versus incidence angle of 633 nm laser beam. The reflection shows a SPP resonance peak at 74°, which is consistent with our calculation. The baseline of reflection increases for larger incidence angle of laser beam, which can be attributed to the scattering loss from nanoporous structure of aerogel. The aerogel substrate has 50 nm-scale nanoporous structure, resulting in unavoidable scattering effect for optical light. The propagation distance of laser light inside a rectangular aerogel substrate is shorter for larger incidence angle, leading to less scattering loss. The reflected laser lights are also presented in Fig. 3(e–g), for various incidence angles of 68°, 74°, and 78°. The movie for angle-scanning SPP resonance is presented in Supporting Information Movie 1.

### Plasmonic refractive index sensor with air-like aerogel

The capabilities of the SPP resonance based refractive index sensor comprising a silver-coated nanograting surface comprising aerogel or PDMS were explored with analytes of various indices of refraction, which were dropped on the samples. To thoroughly investigate the total reflectance spectra, *R*(*λ*), including the specular and diffuse reflections of the surface-textured samples, we scanned a monochromator coupled to a halogen lamp using an ultraviolet–visible–near-infrared spectrometer (UV3600, Shimadzu Scientific Instruments) with a 60 mm-diameter integrating sphere (MPC-3100).

**Figure 4:**
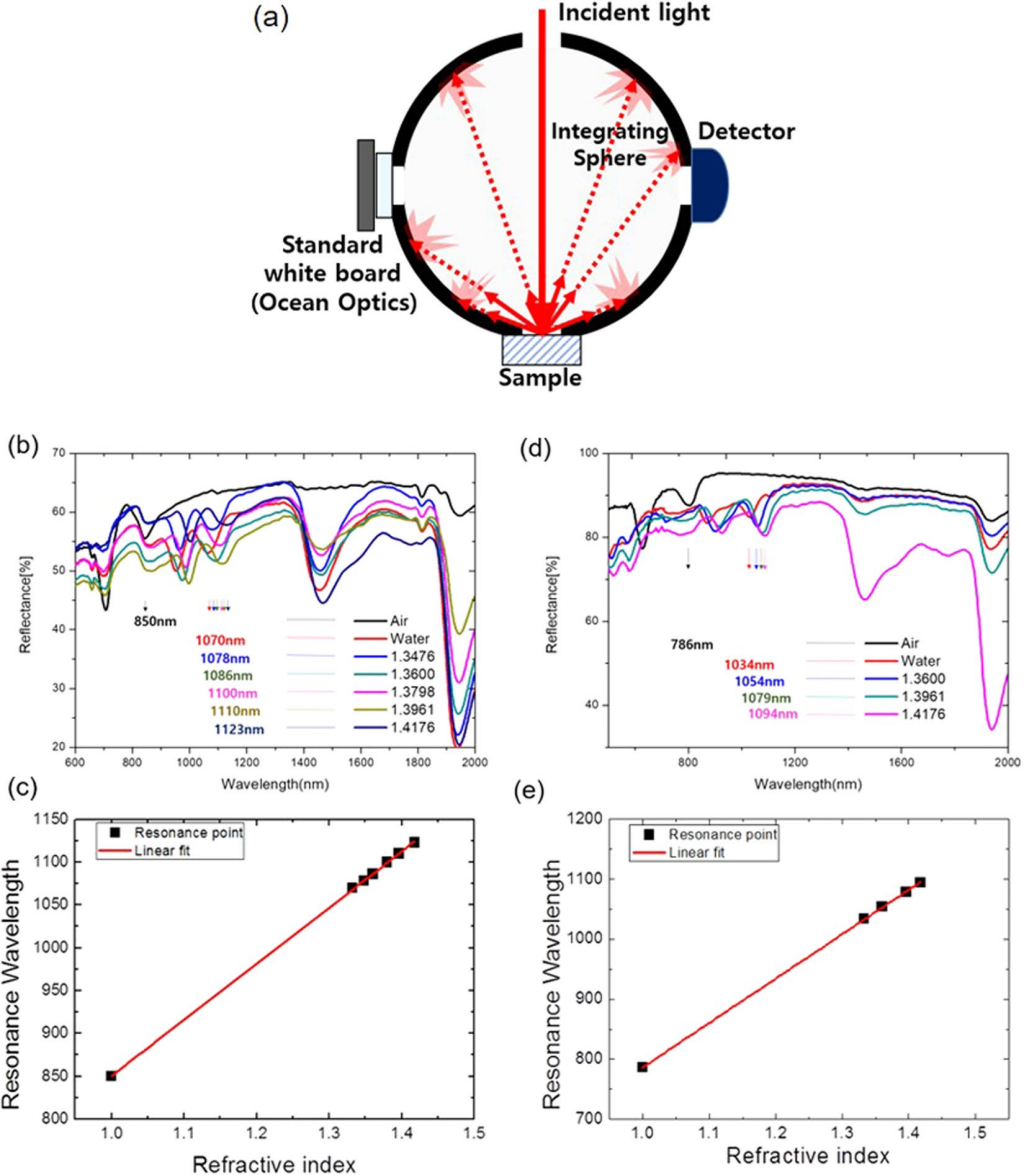
(**a**) The experimental schematic for the spectral measurement with an integrating sphere. (**b**,**d**) Spectral reflectance data for (**b**) PDMS (*n* = 1.51, 7 analytes) and (**d**) aerogel (*n* = 1.074, 5 analytes) substrates with a 60 nm-thick Ag film. (**c**,**e**) Resonance wavelength as a function of analyte refractive index for the (**c**) PDMS and (**e**) aerogel substrates.

For measuring the spectral reflectance data of our samples, an integrating sphere and spectrometer are used and Fig. 4(a) shows the experimental schematic for this measurement. Figure 4(b) shows the reflectance spectra obtained from the silver-coated PDMS substrate with seven analytes of different indices of refraction, while Fig. 4(d) shows that for silver-coated aerogel substrate with five different analytes. It can be seen that the SPP resonance of the aerogel substrate sample was blue-shifted with respect to that of the PDMS substrate. The spectral variations produced by the refractive index variation of the sensing medium are shown in Fig. 4(c,e), which plot the SPP resonance wavelength of the PDMS and aerogel substrates, respectively, versus the analyte refractive index. Interestingly, despite the fact that the peak position of the ultralow-refractive-index substrate is blue-shifted, the sensitivity to the bulk changes of the refractive index, *η*_bulk_, is enhanced, with a value of *η*_bulk_ = 747 and 655 nm/RIU for the aerogel and PDMS, respectively. These experimental results show that the sensitivity of the nanostructure systems can be improved by reducing the refractive index of the substrate. Because the SPP resonance peaks of the aerogel substrate are blue-shifted with respect to those of PDMS, the effective sensitivity includes the factor induced by the peak position of the sensitivity.

### Numerical Simulations with FEM

**Figure 5:**
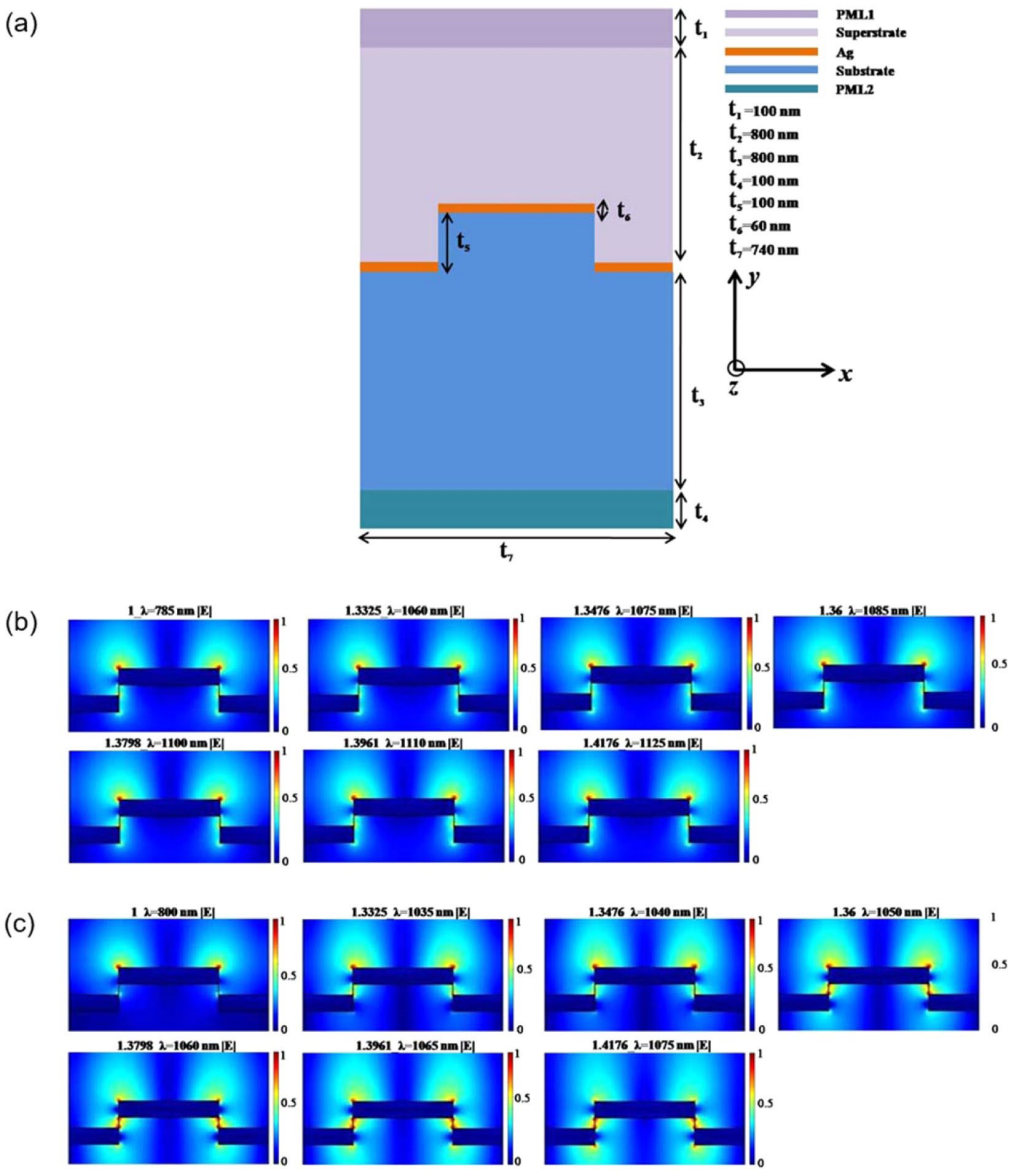
(**a**) Schematic of grating unit cell used for the simulations, where the different layers, their materials and geometric parameters are shown. (**b**,**c**) Simulated surface plots of the norm of the electric field for varying refractive index of the analyte placed on top of silver grating of a (**b**) aerogel and (**c**) PDMS substrate.

We simulated metallic nanograting structure with two different substrates (aerogel, PDMS) to investigate the effect of substrate index on plasmonic refractive index sensing performance. The two-dimensional numerical simulations of the grating structure with various superstrates as analytes (*n* = 1 to 1.4176) and two different substrates (i.e., aerogel, PDMS) were performed using the radio-frequency (RF) module of the COMSOL Multiphysics software based on the finite element method. This elucidated the excitation of the SPP resonances and associated electromagnetic near-fields at the metal-dielectric interfaces. Figure 5(a) shows a schematic of the unit cell of a typical grating structure, showing the various layers and materials involved and their geometric parameters. A wave port boundary condition was used to excite the structure by a transverse-magnetic electromagnetic plane wave launched from the top of the structure at normal incidence. To avoid unwanted reflections and transmissions, perfectly matched layers (PML1, PML2 in Fig. 5(a)) at the top and bottom edges were used, as well as a scattering boundary condition at the bottom edge of the model along the plane wave propagation direction (*y*-axis). To consider the structure as an infinite array, the unit cell was simulated using a perfect electric conductor boundary condition in the transverse direction (*x*-axis) of the plane wave propagation. Triangular mesh conditions were used owing to the thin-film nature of the structure, and the wavelength-dependent reflection of light in a wavelength range of 300–1950 nm was calculated. The dispersive dielectric permittivity of silver was taken from Johnson and Christy’s experimental observations^32^ and was tabulated after interpolation in the COMSOL model.

Excitation of SPP resonances at the metal/dielectric interfaces strengthened the localized electromagnetic near-field. The coupling strength of the near fields into the dielectric medium accounted for the sensitivity of the SPP resonances along with the change in spectral response with the varying analytes. To further understand the behavior of the confined plasmonic near-field in sensing with the varying dielectric substrate, we monitored the effect of the superstrate analytes on the norm of the electric field. The surface plots of the norm of the electric field are shown in Fig. 5(b,c) for the aerogel and PDMS substrates, respectively, where the caption atop each figure shows the refractive index of the superstrate analyte and the corresponding SPP resonance wavelength. For the aerogel substrate (Fig. 5(b)), the distribution of the electric field in the air analyte (upper left image in Fig. 5(b)) is homogeneously localized at the edges of the top ridge along with the corners of the bottom valleys of the grating. Because the refractive indices of the aerogel and air are nearly equivalent, the surface plasmon near field is equally coupled to both the dielectric medium surrounding the metal surface. As we increase the refractive index of the medium, the near-field distribution changes slightly and is seen to be coupled to the aerogel substrate with larger length of distribution in valley while the field on the top ridge decreases. The change in the near-field distribution is appreciable, as is the effect to the sensitivity, as the coupling of the field in the analyte is reduced. A similar trend is visible in all cases when the refractive index increases further. In case of *n* = 1.4176 analyte (bottom right image in Fig. 5(b)), the coupling of the field into the substrate is high and the field on the top ridges is weaker; however, the change of the field in the analyte is very moderate and the field strength changes very slightly, which makes the surface plasmon sensing still comparable to that in the case of *n* = 1 analyte.

To understand the effect of the substrate medium on the plasmonic near-field, and thus its effect on the sensitivity of the SPP resonances, we performed a similar numerical simulation for a grating structure with a PDMS substrate. As can be seen in the surface plots of the norm of electric field shown in Fig. 5(c), the near field of the air analyte (upper left image in Fig. 5(c)) is mostly localized on the top of the ridge and the coupling of the near-field in the corners of the valley to the air analyte is insignificant. This coupling of the near-field to an analyte with a lower refractive index corresponded with the observation in the case of an aerogel substrate. As the refractive index of analyte increases, the near-field coupling to the substrate medium become appreciable and the field becomes localized in the corners of the valley along with the top ridge of the grating structure. The field strength on the edges of the top ridge decreases as the analyte refractive index increases. In case of the analyte with *n* = 1.3798 (lower left image in Fig. 5(c)), the substrate refractive index (*n* = 1.4) is nearly equivalent to that of the analyte. Hence, the field on the top ridge is seen to couple with the field in the valley and exhibits a distribution similar to that of the air analyte with the aerogel substrate (upper left image in Fig. 5(b)). The field strength is almost the same in both media at the metal interface. As the analyte refractive index is further increased to *n* = 1.4176 (bottom right image in Fig. 5(c)), the field couples to the PDMS substrate with the least amount of confinement on the top ridge in the analyte. This is somewhat the reverse of the result observed for the case of aerogel substrate, where the field strength in the analyte is high for *n* = 1.4176. This simulation study shows the explicit coupling and localization of the surface plasmon near-field with the two different substrate media with varying analytes, where the near-field favors localization in the low-index material at the metal interface. This behavior of the near-field distribution accounts for the higher sensitivity of the aerogel substrate compared to that of the PDMS, considering that the surface plasmon field penetration into the analyte is higher with the aerogel substrate, resulting in a more effective way to interact with the analyte medium.

**Figure 6:**
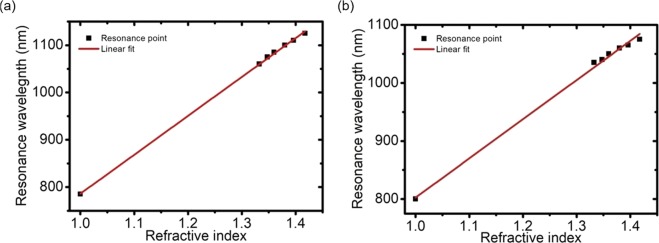
Simulated surface plasmon resonance wavelength vs. analyte refractive index used with an (**a**) aerogel and (**b**) PDMS substrate.

The ultralow-index of the aerogel substrate shifts the optical fields from the substrate to the analyte side, where the stronger optical field in the analyte side leads to the enhancement of the sensitivity of the ultralow-index substrate for plasmonic platforms. The reflection spectra for these simulated samples were calculated, and the SPP resonance wavelengths were plotted as a function of the analyte refractive index for the aerogel (Fig. 6(a)) and PDMS (Fig. 6(b)). The calibrated slopes show that the refractive index sensitivities are 822.9 and 674.5 nm/RIU for the aerogel and PDMS substrates, respectively. These results show that the use of an aerogel substrate provides significant benefits for plasmonic sensing.

## Conclusion

In this paper, we proposed a novel ultralow-index substrate for plasmonic sensing applications. Two samples with two different substrate materials of an aerogel (*n* = 1.08) and PDMS (*n* = 1.40) possessing different refractive indices were fabricated using the imprinting method, whereupon a one-dimensional grating structure was patterned. An experimentally-observed angular SPP resonance dip at 74° was found for the ultralow-index aerogel substrate, which was consistent with theory. The SPP resonance wavelength was measured at the interface of the metal grating and the aerogel and PDMS substrates, where the change in resonance wavelength with the varying refractive index of the superstrate analyte was more prominent for the ultralow-index substrate (i.e., aerogel) than the higher-index substrate (i.e., PDMS). The observed value of Δ*λ*/Δ*n* for the aerogel and the PDMS were 740.2 and 655.9 nm/RIU, respectively. The numerical simulation revealed that the low-index substrate enabled coupling of the surface plasmon near-field well into the analyte, thus making it a good candidate for sensing application. The experimentally-observed SPP resonance wavelengths for the two substrate materials with varying analytes atop the metal grating structure correlated well with the simulated results.

## Supplementary information

Supplementary information_SREP-18-33303

Supporting information movie1

## References

[1] Bahraminpanah M, Abrishamian MS, Mirtaberi SA, Liu J-M (2014). Ultracompact Plasmonic Loop–Stub Notch Filter and Sensor. *Sens. Actuators*. B..

[2] Hiep HM, Yoshikawa H, Tamiya E (2010). Interference Localized Surface Plasmon Resonance Nanosensor Tailored for the Detection of Specific Biomolecular Interactions. Anal. Chem..

[3] Hotta K, Yamaguchi A, Teramae N (2012). Nanoporous Waveguide Sensor with Optimized Nanoarchitectures for Highly Sensitive Label-Free Biosensing. ACS Nano..

[4] Ai B, Yu Y, Möhwald H, Zhang G (2013). Responsive Monochromatic Color Display Based on Nanovolcano Arrays. Adv. Opt. Mater..

[5] Dou Y (2012). Fabrication of MMO–TiO2 one-dimensional photonic crystal and its application as a colorimetric sensor. J. Mater. Chem..

[6] Khorasaninejad M, Abedzadeh N, Walia J, Patchett S, Saini SS (2012). Color Matrix Refractive Index Sensors Using Coupled Vertical Silicon Nanowire Arrays. Nano Lett..

[7] Khorasaninejad M (2013). Colorimetric Sensors Using Nano-Patch Surface Plasmon Resonators. Nanotechnology..

[8] Homola J (2008). Surface Plasmon Resonance Sensors for Detection of Chemical and Biological Species. Chem. Rev..

[9] Estevez M-C, Otte MA, Sepulveda B, Lechuga LM (2014). Trends and Challenges of Refractometric Nanoplasmonic Biosensors. Anal. Chim. Acta..

[10] Fang J (2015). Plasmonic Metamaterial Sensor with Ultra-High Sensitivity in the Visible Spectral Range. Adv. Opt. Mater..

[11] Strobbia, P., Languirand, E. R., Cullum, B. M. Recent Advances in Plasmonic Nanostructures for Sensing: A Review. *Opt. Eng*. **54**, 10, 100902 (2015).

[12] Lim LK, Ng BK, Fu CY, Tohing LY (2017). Highly Sensitive and Scalable AAO-Based Nano-fibre SERS Substrate for Sensing Application. Nanotechnology..

[13] Hao Q (2017). Facile Design of Ultra-Thin Anodic Aluminum Oxide Membranes for the Fabrication of Plasmonic Nanoarrays. Nanotechnology..

[14] Gupta P, Ramakrishna SA, Wanare H (2016). Strong coupling of surface plasmon resonances to molecules on a gold grating. Journal of Optics..

[15] Gupta, Prince. Controlling Level Splitting by Strong Coupling of Surface Plasmon Resonances with Rhodamine-6G on a Gold Grating. *Plasmonics*. 1–11. (2018).

[16] Brian B, Sepúlveda B, Alaverdyan Y, Lechuga LM, Käll M (2009). Sensitivity Enhancement of Nanoplasmonic Sensors in Low Refractive Index Substrates. Opt. Express..

[17] Kang G, Yoo J, Ahn J, Kim K (2015). Transparent Dielectric Nanostructures for Efficient Light Management in Optoelectronic Applications. Nano Today..

[18] Shubert EF, Kim JK, Xi JQ (2007). Low-Refractive-Index Materials: A New Class of Optical Thin-FilmMaterials. Phys. Stat. Solid B..

[19] Walheim S, Schäffer E, Mlynek J, Steiner U (1999). Nanophase-Separated Polymer Films as High-Performance Antireflection Coatings. Science..

[20] Wang NF (2012). Porous SiO/MgF Broadband Antireflection Coatings for Superstrate-Type Silicon-Based Tandem Cells. Opt. Express..

[21] Mark, J. E. *Polymer Data Handbook*; Oxford University Press: New York, (1999).

[22] Chattopadhyay S, Huang YF, Jen YJ, Ganguly A, Chen KH (2010). Anti-Reflecting and Photonic Nanostructures. Mater. Sci. Eng., R..

[23] Chen DA-R (2001). (AR) Coatings Made by Sol-Gel Processes: A Review. Sol. Energy Mater. Sol. Cells..

[24] Nakanishi K, Tanaka N (2007). Sol-Gel with Phase Separation. Hierarchically Porous Materials Optimized for High-Performance Liquid Chromatography Separations. Acc. Chem. Res..

[25] Kanamori K, Aizawa M, Nakanishi K, Hanada T (2007). New Transparent Methylsilsesquioxane Aerogels and Xerogels with Improved Mechanical Properties. Adv. Mater..

[26] Buzykaev AR, Danilyuk AF, Ganzhur SF, Kravchenko EA, Onuchin AP (1999). Measurement of Optical Parameters of Aerogel. Nucl. Instr. Meth. Phys. Res. A..

[27] Shin D (2017). Scalable Variable-Index Elasto-Optic Metamaterials for Macroscopic Optical Components and Devices. Nat. Comm..

[28] Kim, C. *et al*. Large-Scale Nanoporous Metal-Coated Silica Aerogels for High Raman Gain, submitted. 10.1038/s41598-018-33539-z PMC6181977 30310142

[29] Bae K (2015). Refractometric and Colorimetric Index Sensing by a Plasmon-Coupled Hybrid AAO Nanotemplate. RSC Adv..

[30] Lee J (2015). Photonic Crystal Heterostructure for High Q Refractive Index Sensing. RSC Adv..

[31] Palik, E., Ghosh, G. *Handbook of Optical Constants of Solids, Vol. 2*, 1st ed; Academic Press: San Diego (1999).

[32] Johnson PB, Christy RW (1972). Optical Constants of the Noble Metals. Phys. Rev. B..

