# Supplementary material for: Air-like plasmonics with ultralow-refractive-index silica aerogels
Source: Sci Rep. 2019 Feb 19;9:2265. doi: 10.1038/s41598-019-38859-2 (PMC6381121; doi:10.1038/s41598-019-38859-2)
Supplement: Supplementary file 1 — Supplementary information_SREP-18-33303 [file 41598_2019_38859_MOESM1_ESM.docx]

Supplementary Information

Air-like plasmonics with ultralow-refractive-index silica aerogels

Yeonhong Kim^1†^, Seunghwa Baek^1†^, Prince Gupta^1^, Changwook Kim^1^, Kiseok Chang^2^, Sung-Pil Ryu^2^, Hansaem Kang^2^, Wook Sung Kim^2^, Jaemin Myoung^3^, Wounjhang Park^4^, and Kyoungsik Kim^1*^

^1^ School of Mechanical Engineering, Yonsei University, 50 Yonsei-ro, Seodaemun-gu, Seoul 03722, Republic of Korea.

^2^ Technology Collaboration Team, LG Display Co., Ltd., Gyeonggi-do 413-811, Republic of Korea

^3^ Department of Materials Science and Engineering, Yonsei University, Seoul 03722, Republic of Korea

^4^ Department of Electrical Engineering, University of Colorado, Boulder, Colorado, USA

^†^ These authors contributed equally to this work.

^*^Correspondence and requests for materials should be addressed to K.K. (email: [kks@yonsei.ac.kr](mailto:kks@yonsei.ac.kr)).


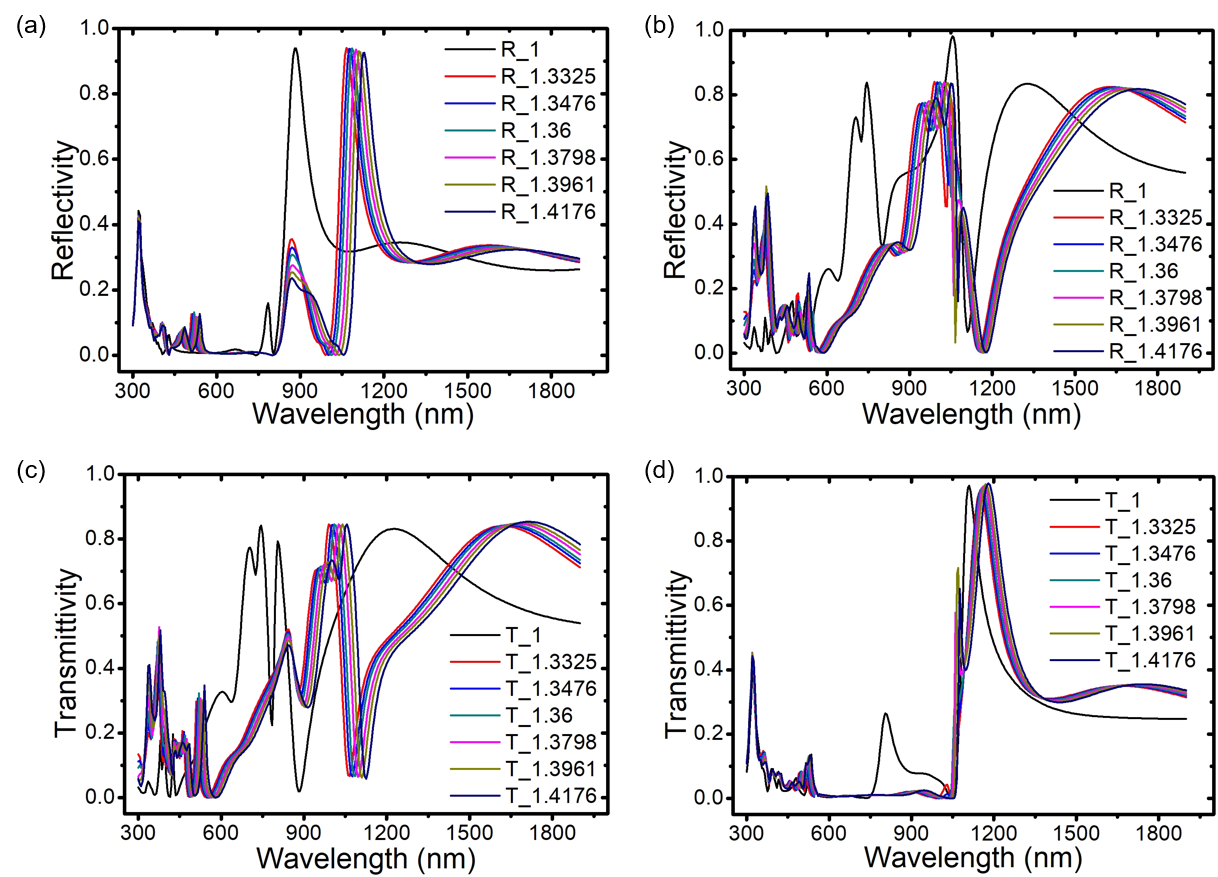


**Figure S1.** The calculated reflaection spectra of (a) aerogel and (b) PDMS. The calculated transmission spectra of (c) aerogel and (d) PDMS.


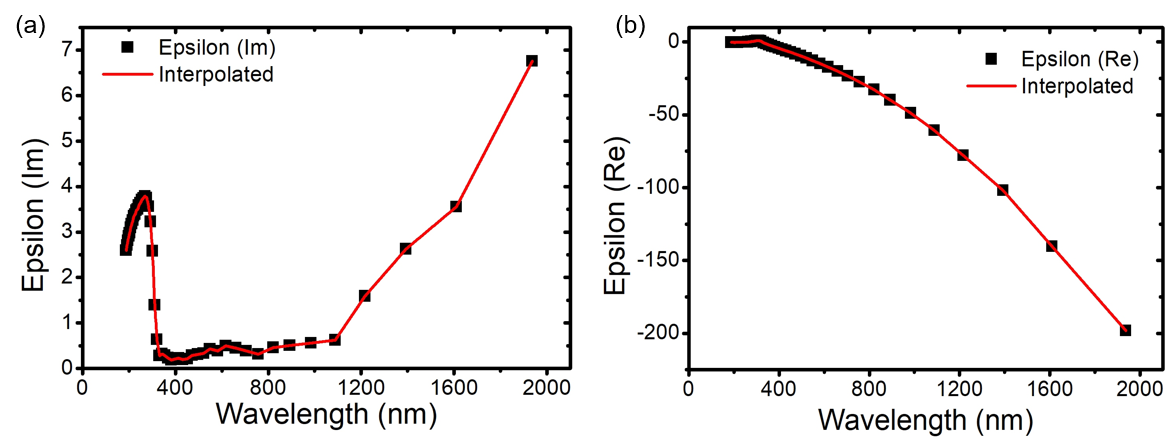


**Figure S2.** The dispersion effect of silver in (a) imaginary, and (b) real parts in permittivity.
